# Clinical and microbiologic features of *Achromobacter* species: a 10-year, multicenter experience

**DOI:** 10.1128/jcm.00724-25

**Published:** 2025-09-19

**Authors:** David R. Bayless, Mitchell G. Dumais, Jack W. McHugh, Nischal Ranganath, Madiha Fida, Supavit Chesdachai, Omar M. Abu Saleh

**Affiliations:** 1 Department of Internal Medicine, Mayo Clinic College of Medicine & Science 198509 , Rochester, Minnesota, USA; 2 Department of Medicine, Division of Public Health, Infectious Diseases, and Occupational Medicine, Mayo Clinic College of Medicine & Science 12270 , Rochester, Minnesota, USA; Children's Hospital Los Angeles, Los Angeles, California, USA

**Keywords:** *Achromobacter*, non-bloodstream *Achromobacter *isolates, *Achromobacter *bloodstream infection, *Achromobacter xylosoxidans*, multicenter study

## Abstract

*Achromobacter* species are Gram-negative bacilli that predominantly affect individuals with cystic fibrosis or those with immunocompromising conditions. Clinical and microbiologic data on *Achromobacter* species are limited due to the rarity of these organisms and challenges with species-level identification. We conducted a 10-year retrospective analysis (1 January 2013 to 14 March 2023) of all bloodstream and non-bloodstream *Achromobacter* isolates identified at three tertiary-care Mayo Clinic locations (Minnesota, Arizona, and Florida). Additionally, we examined clinical characteristics of adult patients with *Achromobacter* bloodstream isolates. A total of 1,598 *Achromobacter* isolates, encompassing 1,545 non-bloodstream isolates and 53 bloodstream isolates, were identified. The most frequently identified species was *Achromobacter xylosoxidans*, though species-level identification was not possible for many isolates. Among adult patients with *Achromobacter* bloodstream infection, the mean age was 58.3 years (standard deviation ± 16.7), 73.1% had a central venous catheter, and 59.6% were immunocompromised. All-cause mortality was 10.2% at 30 days and 18.4% at 90 days. Bloodstream isolates exhibited greater than 90% susceptibility to meropenem, piperacillin-tazobactam, and trimethoprim-sulfamethoxazole. The respiratory tract was the most common source of non-bloodstream isolates (57.5%). Non-bloodstream isolates showed high susceptibility to imipenem (94.6%), piperacillin-tazobactam (92.8%), trimethoprim-sulfamethoxazole (92.1%), and meropenem (87.5%) while demonstrating significant resistance to fluoroquinolones and aminoglycosides. Our findings support the use of piperacillin-tazobactam, carbapenems, or trimethoprim-sulfamethoxazole as initial therapy for *Achromobacter* infections. Further research is needed to better define the clinical and microbiologic characteristics of *Achromobacter* infections.

**IMPORTANCE:**

*Achromobacter* is a rare but important genus of bacteria that tends to cause infection in those with cystic fibrosis, recurrent healthcare exposures, and/or an immunocompromising condition. There is limited data on the clinical profile of *Achromobacter* infections as well as optimal antibiotic selection for affected patients. We conducted a retrospective study to improve understanding of the microbiologic and clinical characteristics of *Achromobacter*. Our study consists of a 10-year survey of all *Achromobacter* isolates processed by three Mayo Clinic tertiary-care centers in Minnesota, Florida, and Arizona from 2013 to 2023. We report multiple findings, including the clinical characteristics of patients with *Achromobacter* bloodstream infection, the number of different *Achromobacter* species identified, and the sources of isolates not obtained from blood cultures. We additionally present antimicrobial susceptibility results for *Achromobacter* isolates. Our study provides guidance to clinicians treating *Achromobacter* infections and, it is hoped, will facilitate ongoing study of *Achromobacter*.

## INTRODUCTION

*Achromobacter* is a genus of aerobic, non-fermenting, Gram-negative bacilli found in both natural and domestic environments (1, 2). More than 20 species (spp.) have been identified, with *Achromobacter xylosoxidans* being the most frequently implicated in human infections (3). Other clinically relevant species include *A. insolitus, A. ruhlandii, A. suavis, A. denitrificans*, and *A. mucicolens* (1, 3, 4). Although *Achromobacter* species are rare, they are increasingly recognized as potential opportunistic pathogens, particularly in patients who are immunocompromised (5).

Interest in *Achromobacter* has increased due to its association with pulmonary infections and colonization in patients with cystic fibrosis (CF), where it is regarded as an emerging pathogen (2–4, 6–9). In CF, chronic *Achromobacter* infection or colonization has been linked to worse clinical outcomes, including accelerated decline in pulmonary infection and higher mortality (5, 10–13). Outside the CF setting, *Achromobacter* infections predominantly affect individuals with immunocompromise but can also occur in immunocompetent hosts (5, 14). The most common clinical presentations include pneumonia and bloodstream infections (BSIs), although other presentations, such as with endocarditis, urinary tract infections, central nervous system infections, ocular infections, and peritonitis, have also been reported (14–19).

Many *Achromobacter* infections are healthcare-associated, with intravenous catheter use frequently implicated in their pathogenesis (8, 14, 16, 18, 20, 21). Outbreaks have been linked to contamination of medical equipment, indirect patient contact, and inadequate sanitation, highlighting the importance of infection control practices (22–25). Long-term data on mortality associated with *Achromobacter* infections are limited, with most evidence derived from small case series (16, 18, 20).

Resistance mechanisms in *Achromobacter* include multidrug efflux pumps and β-lactamase enzymes (8, 13). These and other resistance pathways confer intrinsic resistance to most cephalosporins, certain penicillins, and aminoglycosides and result in variable resistance to tetracyclines and the respiratory fluoroquinolones levofloxacin and moxifloxacin (8, 13). *Achromobacter* can also acquire additional resistance during treatment (8, 13). Given the relative rarity of *Achromobacter* infections, clinical data to guide empiric antibiotic selection remain sparse. However, on the basis of case series and antibiotic susceptibility testing (AST), piperacillin-tazobactam, carbapenems, ceftazidime, and trimethoprim-sulfamethoxazole (TMP-SMX) have been suggested as first-line therapies (8, 13).

The Clinical and Laboratory Standards Institute (CLSI) does not provide genus-specific breakpoints for *Achromobacter* species; however, in clinical practice, the CLSI breakpoints designated for “Other non-Enterobacterales” (non-fastidious, glucose-nonfermenting, Gram-negative bacilli) are commonly used for interpreting antimicrobial susceptibility results of *Achromobacter* isolates. The European Committee on Antimicrobial Susceptibility Testing (EUCAST) provides minimum inhibitory concentration (MIC) breakpoints for *A. xylosoxidans* for piperacillin-tazobactam, meropenem, and TMP-SMX (26). Cefiderocol also appears in the EUCAST *A. xylosoxidans* table, yet its susceptible and resistant columns are left blank; users are directed to the dedicated EUCAST broth-microdilution guidance for cefiderocol released in January 2024, and there is noted to be insufficient clinical data to determine clinical breakpoints (27). Notably, susceptibility breakpoints for *Achromobacter* have remained inconsistent across studies (8, 28). Recent research has been conducted to establish MIC breakpoints for *Achromobacter* species (29).

To better characterize the clinical and microbiologic features of *Achromobacter* spp., we undertook a multi-center evaluation of *Achromobacter* infections drawn from a decade of practice. Study objectives include presentation of *Achromobacter* isolate species and source, clinical characteristics and outcomes of patients with *Achromobacter* bloodstream infection, and antimicrobial susceptibility results for non-bloodstream and bloodstream isolates as well as for pulmonary isolates of *A. xylosoxidans*.

## MATERIALS AND METHODS

We retrospectively assessed all *Achromobacter* culture isolates obtained within a 10-year period (1 January 2013 to 14 March 2023) at the reference laboratories at three Mayo Clinic tertiary care centers located in Rochester, Minnesota (MN); Jacksonville, Florida (FL); and Phoenix, Arizona (AZ).

### Data collection

Data from medical record reviews were stored securely online using the Research Electronic Data Capture (REDCap) application. The study was exempt from patient consent as it did not involve patient contact or experimentation. The study was approved by the Mayo Clinic Institutional Review Board (#23-002594).

### Microbiology and antimicrobial susceptibility testing

Bloodstream and non-bloodstream isolates were evaluated separately. Blood cultures were processed using the BD BACTEC FX system (Becton Dickinson, Franklin Lakes, NJ), with each set comprizing one anaerobic bottle and two aerobic bottles. Cultures were incubated for up to five days per standard protocol. Non-bloodstream samples were collected and processed according to standard operating procedures based on anatomic source and specimen type.

*Achromobacter* species were identified using matrix-assisted laser desorption/ionization time-of-flight mass spectrometry (MALDI-TOF MS). MALDI-TOF MS was implemented at our institution in 2012 and became the primary method for genus- and species-level identification. Prior to its use, identification relied on biochemical assays including oxidase positivity, weak glucose oxidation, nitrate reduction with gas production, and motility. Additional testing with selective media, such as MacConkey and Cetrimide agar, supported differentiation from other non-fermenting Gram-negative bacilli. While MALDI-TOF MS allowed reliable genus-level identification, species-level resolution was occasionally limited by database constraints, particularly when differentiating among closely related species. When MALDI-TOF MS was inconclusive, 16S rRNA gene sequencing was used for further classification. When species-level identification was not possible, isolates were reported as “*Achromobacter* sp.,” “*Achromobacter*,” or “*Achromobacter* XX/XX/XX,” where “XX” refers to two or more possible species names. For this study, all such cases were categorized as “*Achromobacter* sp.” for analysis.

Phenotypic antimicrobial susceptibility testing (AST) of *Achromobacter* isolates was performed using the CLSI reference agar dilution method. Mueller-Hinton agar was used with a standardized inoculum. Plates were incubated at 35°C in ambient air, and results were read at 16–20 h. MIC interpretations followed CLSI standards for “Other non-Enterobacterales” (30). No changes to CLSI MIC breakpoints were made during the study period (29, 30). Tigecycline testing was performed using E-test (bioMérieux, Durham, NC).

Routine AST was performed for amikacin, aztreonam, cefepime, ceftazidime, ciprofloxacin, gentamicin, levofloxacin, meropenem, piperacillin-tazobactam, tobramycin, and TMP-SMX. Additional agents, such as colistin, imipenem, minocycline, and doxycycline, were tested at the request of the clinical team or when clinically indicated.

### Bloodstream isolates

Retrospective medical record review was conducted for adult patients diagnosed with *Achromobacter* BSI. Data collection included patient demographics, past medical history and hospitalization status, recent antibiotic exposure, Charlson Comorbidity Index (CCI), Pitt bacteremia score, source of BSI, monomicrobial vs polymicrobial BSI status, antibiotics used for definitive therapy, and clinical outcomes (31). Patients who did not provide research authorization were excluded.

When two or more *Achromobacter* isolates were identified in the same patient at the time of initial positive blood cultures, they were regarded as the same *Achromobacter* isolate unless they had different AST profiles and were obtained from blood cultures on the same day, or if they clearly belonged to different species (e.g., *A. xylosoxidans* and *A. insolitus*). When there was ambiguity with species-level identification (e.g., two isolates reported as *Achromobacter* XX/XX/XX and *A. xylosoxidans*, respectively)*,* isolates were classified as one “*Achromobacter* sp.” isolate.

Neutropenia was defined as an absolute neutrophil count of <500 cells/µL within 48 h of BSI onset. Polymicrobial BSI was defined as co-infection with a non-*Achromobacter* bloodstream pathogen within 7 days of *Achromobacter* BSI. Co-infecting pathogen(s) were recorded in cases of polymicrobial BSI.

The presumed source of BSI was determined based on documentation from infectious disease specialists and/or the primary hospital team. If no clear source was identified, the BSI source was listed as unknown. Potential infection sources were still listed in certain cases of BSI of unknown source if diagnostic evaluation was limited, or if the clinical significance of the potential infection sources was not determined. BSI relapse was defined as growth of the same organism in blood culture within 12 weeks following documented clearance of the initial infection. For deceased patients, two independent reviewers (D.B. and J.M.) assessed whether death was attributable to *Achromobacter* BSI. Death was attributed to *Achromobacter* BSI only if both reviewers concurred.

### Non-bloodstream isolates

Non-bloodstream isolates were grouped into quartiles based on collection date (2013–2015, 2016–2018, 2019–2021, and 2022–2023). The specimen source and AST results were recorded. Susceptibility data were reported in aggregate for all isolates, as well as for pulmonary isolates of *A. xylosoxidans*. Exclusion criteria included patients under 18 years of age without parental or legal guardian consent and adult patients who did not provide research authorization.

### Statistical analysis

Clinical and outcome data were summarized using means or medians for continuous variables and percentages for categorical variables. Categorical comparisons, including resistance rates between sources, were assessed using Chi-square tests where appropriate. *P*-value < 0.05 was regarded as significant. Statistical analysis was performed using Microsoft Excel (Excel 2024, Microsoft Corporation, Redmond, USA).

## RESULTS

### Bloodstream infections

Fifty-two cases of *Achromobacter* bloodstream infection (BSI) were identified during the 10-year study period, encompassing 53 unique bloodstream isolates. The mean age of patients was 58.3 years (SD, ±16.7; Table 1). The median CCI was 5 (IQR, 3–8), median hospital length of stay was 11 days (IQR 5.3–21.8), and 11 (21.2%) patients were admitted to the intensive care unit (ICU) within 24 h of diagnosis (Table 1). Thirty-one patients (59.6%) were immunocompromised, most commonly due to hematopoietic stem cell transplantation (23.1%). Neutropenia was present in 10 (19.2%) cases.

**TABLE 1:** Clinical characteristics of patients with *Achromobacter* bloodstream infection^*a*^

Variable	Data (*n* = 52)^*b*^
Age (mean [SD] [yrs])	58.3 (16.7)
Male sex (n [%])	28 (53.8)
Race/ethnicity (n [%])	
White	41 (78.8)
Black or African American	6 (11.5)
Asian or Asian Filipino	3 (5.8)
Hispanic	2 (3.8)
Charlson Comorbidity Index (median [IQR])	5 (3–8)
Pitt bacteremia score (n [%])	
0	36 (69.2)
1	2 (3.8)
2	8 (15.4)
3	2 (3.8)
4–6	4 (7.7)
Hospital LOS (median [IQR] [days])	11 (5.3–21.8) (*n* = 48)
ICU admission within 24 h of BSI diagnosis (n [%])	11 (21.2)
ICU length of stay (median [IQR])	15 (1-45) (*n* = 11)
History of end-stage liver disease/cirrhosis (n [%])	8 (15.4)
History of end-stage renal disease requiring dialysis (n [%])	8 (15.4)
History of CF (n [%])	2 (3.8)
History of non-CF structural lung disease or tracheostomy dependence^*c*^ (n [%])	6 (11.5)
History of type one or two diabetes mellitus (n [%])	12 (23.1)
Obesity with BMI >30 kg/m^2^ (n [%])	17 (32.7)
Presence of a central venous catheter (n [%])	38 (73.1)
Invasive procedure within 30 days prior to BSI^*d*^	25 (48.1)
Non-ICU hospital admission within 30 days prior to admission (n [%])	20 (42.6) (*n* = 47)
ICU hospital admission within 30 days prior to admission (n [%])	9 (19.1) (*n* = 47)
Antibiotic exposure within 30 days prior to BSI (n [%])	36 (70.6) (*n* = 51)
Immunocompromising condition (n [%])	31 (59.6)
Hematopoietic stem cell transplant	12 (23.1)
Hematologic malignancy	8 (15.4)
Solid organ transplant	5 (9.6)
Solid organ malignancy	4 (7.7)
Other	2 (3.8)
Immunocompromising medication use at time of BSI diagnosis (n [%])^*e*^	16 (30.8)
Neutropenia with ANC < 500 cells/µL within 48 h of BSI diagnosis (n [%])	10 (19.2)
Blood culture collection site (with growth of *Achromobacter* sp.) (n [%])	
Peripheral collection	31 (59.6)
Central venous catheter	11 (21.2)
Both	10 (19.2)
Time to clearance of blood cultures (mean [SD] [days])	2.96 (1.9) (*n* = 46)
*Achromobacter* blood culture isolate (n [%])	
*A. xylosoxidans*	33 (63.5)
*A. insolitus*	1 (1.9)
*Achromobacter* sp. (species unidentified)	18 (34.6)
Potential source of BSI (n [%]; multiple sources allowed)	
Vascular catheter-related	30 (57.7)
Respiratory	11 (21.2)
Intra-abdominal (non-urinary/prostatic)	11 (21.2)
Skin and soft tissue	5 (9.6)
Urinary tract	3 (5.8)
Bone and joint	1 (1.9)
Implant/hardware	1 (1.9)
Endovascular	1 (1.9)
Unknown/could not be determined	7 (13.5)
Polymicrobial BSI (n [%])	25 (48.1)
Antibiotics used for treatment based on AST results (n [%])^*f*^	
Carbapenems	16 (34.8) (*n* = 46)
Piperacillin-tazobactam	11 (23.9)
TMP-SMX	11 (23.9)
Fluoroquinolones	10 (21.7)
Tigecycline	1 (2.2)
Other beta-lactam antibiotic	2 (4.3)
Duration of antibiotic therapy (median [IQR], days)	16 (13-21) (*n* = 41)
All-cause 30-day mortality (n [%])	5 (10.2) (*n* = 49)
All-cause 90-day mortality (n [%])	9 (18.4) (*n* = 49)
Death attributable to *Achromobacter* BSI (n [%])	4 (7.7)
Microbiologic relapse (n [%])	1 (2.0) (*n* = 49)
Number of BSI isolates with multidrug resistance^*g*^ (n [%]):	
Patients admitted to ICU within 24 h of BSI	9 (90) (*n* = 10)
Patients not admitted to ICU within 24 h of BSI	37 (88.1) (*n* = 42)
Patients deceased within 30 days of BSI	5 (100) (*n* = 5)
Patients not deceased within 30 days of BSI	41 (87.2) (*n* = 47)

^*a*^ IQR = inter-quartile range; LOS = length of stay; ICU = intensive care unit.

^*b*^ If data for a certain variable were not available for all 52 cases, the number of cases with available data is indicated in brackets.

^*c*^ Includes chronic obstructive pulmonary disease, bronchiectasis, and pulmonary fibrosis.

^*d*^ Includes any documented non-dental invasive procedure, from major surgery to more minor procedures, such as central venous catheter placement or bone marrow biopsy.

^*e*^ Includes chemotherapy, glucocorticoids, disease-modifying antirheumatic agents, and other immunosuppressing medications.

^*f*^ Includes patients who received combination antibiotic therapy. From available information, antibiotic therapy was not pursued in five cases. In two cases, treatment consisted of central venous catheter removal alone; in one case, the patient was transitioned to comfort cares; in one case, *Achromobacter *blood culture result was treated as a contaminant; in the final case, there was no clear evidence that *Achromobacter *bloodstream infection was treated. In one case, the patient was referred to a local hospital for treatment of *Achromobacter *BSI, but details of the treatment plan are not available.

^*g*^ Multidrug resistance is defined as resistance to three or more classes of antimicrobials.

The predominant bloodstream isolate was *A. xylosoxidans*, which accounted for 33 (63.5%) of 52 cases. The most commonly identified source of BSI was vascular catheter-related (57.7%). Nearly half (48.1%) of cases were polymicrobial. The most common co-infecting pathogens were coagulase-negative staphylococci (Table S1).

The most frequently used antibiotics for definitive therapy were carbapenems (34.8%; *n* = 46), followed by piperacillin-tazobactam and TMP-SMX (23.9% each; Table 1). For patients who were treated with carbapenems, all but three patients were treated with meropenem, and among these three, two were treated with ertapenem and one was treated with imipenem. The median duration of antibiotic therapy was 16 days (IQR, 13–21 days; Table 1). Four patients were treated simultaneously with two agents targeted towards *Achromobacter* as part of their regimen, with regimens consisting of piperacillin-tazobactam plus TMP-SMX, meropenem plus TMP-SMX, levofloxacin plus TMP-SMX, and ceftolozane-tazobactam plus tigecycline. All-cause mortality was 10.2% at 30 days and 18.4% at 90 days (*n* = 49; Table 1). Three patients did not have sufficient follow-up documentation available to determine mortality status at 30 and 90 days. Death was determined to be attributable to *Achromobacter* BSI in four cases.

### Antimicrobial susceptibility testing (bloodstream isolates)

Susceptibility results for bloodstream isolates are presented in Table 2. Susceptibility exceeded 90% for meropenem, piperacillin-tazobactam, and TMP-SMX. Resistance was high for aminoglycosides (tobramycin, amikacin, gentamicin), aztreonam, and ceftriaxone, with more than 75% of isolates demonstrating resistance to these agents. When analyzed by individual species, susceptibility to meropenem, piperacillin-tazobactam, and TMP-SMX remained high (>90%) for *A. xylosoxidans* and *Achromobacter* sp. isolates (Table 3).

**TABLE 2:** Antibiotic susceptibility testing results for *Achromobacter* bloodstream isolates during the study period^*a*^

Antibiotic	No. of isolates^*c*^	% susceptible (MIC)^*d*^	% intermediate (MIC)	% resistant (MIC)
Amikacin	52	13 (≤16)	8 (32)	79 (≥64)
Aztreonam	40	2.5 (≤8)	2.5 (16)	*95 (≥32)*
Cefepime	52	13.5 (≤8)	26.9 (16)	59.6 (≥32)
Ceftriaxone	10	0 (≤8)	0 (16–32)	100 (≥64)
Ceftazidime	44	**81.8 (≤8)**	6.8 (16)	11.4 (≥32)
Ciprofloxacin	52	11.5 (≤1)	25 (2)	63.5 (≥4)
Ertapenem^*b*^	8	0	0	100
Gentamicin	52	7.7 (≤4)	3.8 (8)	*88.5 (≥16)*
Imipenem	12	83.3 (≤4)	8.3 (8)	8.3 (≥16)
Levofloxacin	42	69 (≤2)	19 (4)	11.9 (≥8)
Meropenem	38	**94.7 (≤4)**	0 (8)	5.3 (≥16)
Piperacillin-tazobactam	52	**92.3 (≤16/4)**	3.8 (32/4-64/4)	3.8 (≥128/4)
Tobramycin	52	7.7 (≤4)	7.7 (8)	*84.6 (≥16)*
TMP-SMX^*e*^	52	**96.2 (≤2/38)**		3.8 (≥4/76)
Doxycycline	1	0 (≤4)	0 (8)	100 (≥16)
Minocycline	1	100 (≤4)	0 (8)	0 (≥16)
Tetracycline	1	0 (≤4)	0 (8)	100 (≥16)

^*a*^ Bold face denotes susceptibility of 80% or greater to the tested antibiotic with at least 30 isolates tested, while Italics text denotes resistance rates of 80% or greater with at least 30 isolates tested. Antibiotics included in the gray cells were not routinely tested.

^*b*^ MIC breakpoints are not provided by the CLSI for “Other non-Enterobacterales” for ertapenem.

^*c*^ Among the 52 cases of *Achromobacter *BSI identified, AST could not be completed for one *Achromobacter xylosoxidans *isolate due to failure of the organism to grow on the testing medium. However, for one BSI case there were two* A. insolitus *isolates identified on the same day and with different AST profiles, so the final number of unique isolates is 52.

^*d*^ MICs reported as mcg/mL. CLSI MIC breakpoints are provided in parentheses.

^*e*^ The CLSI does not provide an MIC breakpoint for intermediate resistance with trimethoprim-sulfamethoxazole (TMP-SMX) for “Other non-Enterobacterales”.

**TABLE 3:** Susceptibility of *Achromobacter* blood culture isolates by *Achromobacter* species

Antibiotic (no. of isolates tested)	No. susceptible isolates/No. isolates tested (% susceptible)		
	*A. xylosoxidans*	*Achromobacter* sp.*^a^*	*A. insolitus^b^*
Amikacin (52)^*c*^	*0/32 (0)*	5/18 (27.8)	2/2 (100)
Aztreonam (40)	0/23 (0)	1/15 (6.7)	0/2 (0)
Cefepime (52)	*1/32 (3.1)*	5/18 (27.8)	1/2 (50)
Ceftriaxone (10)	0/7 (0)	0/3 (0)	
Ceftazidime (44)	20/27 (74.1)	14/15 (93.3)	2/2 (100)
Ciprofloxacin (52)	*2/32 (6.3)*	4/18 (22.2)	0/2 (0)
Ertapenem (8)	0/5 (0)	0/3 (0)	
Gentamicin (52)	*0/32 (0)*	4/18 (22.2)	0/2 (0)
Imipenem (12)	6/7 (85.7)	4/5 (80)	
Levofloxacin (42)	17/25 (68)	10/15 (66.7)	2/2 (100)
Meropenem (38)	20/21 (95.2)	16/17 (94.1)	
Piperacillin-tazobactam (52)	**29/32 (90.6)**	17/18 (94.4)	2/2 (100)
Tobramycin (52)	*0/32 (0)*	4/18 (22.2)	0/2 (0)
TMP-SMX (52)	**31/32 (96.9)**	18/18 (100)	1/2 (50)

^*a*^ Indicates isolates without species identification.

^*b*^ Empty cells indicate the isolate was not tested against the listed antibiotic.

^*c*^ Bold face denotes susceptibility of 80% or greater to the tested antibiotic with at least 30 isolates tested, while Italics text denotes resistance rates of 80% or greater with at least 30 isolates tested.

### Non-bloodstream isolates

A total of 1,545 unique non-bloodstream *Achromobacter* isolates were identified from 1,505 patients. Species-level identification was possible in 46.1% of cases (Table 4). Among identified isolates, *A. xylosoxidans* was the most common species, accounting for 40.1% of isolates (Table 4). The proportion of *Achromobacter* isolates without species identification increased over time, peaking at 61.3% in the final study quartile (2022–2023), while the percentage of *A. xylosoxidans* isolates was lowest during this period (34.8%) (Table 4). A Cochran–Armitage test finds a significant upward trend (Z = 2.9, *P* = 0.004) in the proportion of *Achromobacter* isolates without species identification during the study period. Conversely, the proportion of *A. xylosoxidans* isolates declined from 45.7% (153/335) in 2013–2015 to 34.8 % (88/253) in 2022–2023. A Cochran–Armitage test for trend showed this decrease was also significant (Z = –2.1, *P* = 0.039).

**TABLE 4:** Non-bloodstream *Achromobacter* species isolates stratified by the years samples were collected^*a*^

Achromobacter isolate	2013–2015	2016–2018	2019–2021	2022–2023	Total isolates per species^*c*^
*Achromobacter* sp.^*b*^	153 (45.7)	295 (57.2)	229 (51.9)	155 (61.3)	832 (53.9)
*A. xylosoxidans*	153 (45.7)	196 (38.0)	183 (41.5)	88 (34.8)	620 (40.1)
*A. denitrificans*	15 (4.5)	9 (1.7)	7 (1.6)	1 (0.4)	32 (2.1)
*A. insolitus*	14 (4.2)	14 (2.7)	9 (2.0)	3 (1.2)	40 (2.6)
*A. mucicolens*	0	2 (0.4)	13 (2.9)	6 (2.4)	21 (1.4)
Total isolates per quartile	335	516	441	253	1,545

^*a*^ Percentages of species are listed in parentheses (%). Sample totals by species are listed in the right column, whereas sample totals by quartile of years are listed in the bottom row; 1,545 is the total number of isolates analyzed.

^*b*^ Indicates an isolate without species identification.

^*c*^ Gray cells indicate total numbers of isolates per species or per quartile.

The respiratory tract was the most common source of non-bloodstream isolates (57.5%), which included sputum samples, tracheal aspirates, bronchoalveolar lavage specimens, and respiratory tract tissue (Table 5). Wound and musculoskeletal sources accounted for 16.2% of cases, while genitourinary and ear/nose/throat sources were identified in 12.3% and 10.2% of cases, respectively (Table 5). The distribution of isolate sources remained stable throughout the study period, with respiratory sources consistently the most frequent (Table 5).

**TABLE 5:** Non-bloodstream *Achromobacter* isolates stratified by sample source and years samples were collected^*a*^

	2013–2015	2016–2018	2019–2021	2022–2023	Total isolates per source
Respiratory^*b*^	131 (39.3)	219 (42.7)	197 (44.9)	104 (41.3)	651 (42.4)
Respiratory (bronchoscopy, bronchoalveolar lavage)	53 (15.9)	75 (14.6)	65 (14.8)	40 (15.9)	233 (15.2)
Ear/nose/throat^*c*^	37 (11.1)	46 (9.0)	44 (10.0)	30 (11.9)	157 (10.2)
Lymphatic system	0	0	0	1 (0.4)	1 (0.1)
Heart^*d*^	0	6 (1.2)	2 (0.5)	1 (0.4)	9 (0.6)
Gastrointestinal	4 (1.2)	1 (0.2)	0	0	5 (0.3)
Abdominal^*e*^	8 (2.4)	19 (3.7)	7 (1.6)	1 (0.4)	35 (2.3)
Genitourinary	43 (12.9)	63 (12.3)	49 (11.2)	34 (13.5)	189 (12.3)
Musculoskeletal/Wound	55 (16.5)	80 (15.6)	74 (16.9)	40 (15.9)	249 (16.2)
Eye	1 (0.3)	4 (0.8)	0	1 (0.4)	6 (0.4)
Cerebrospinal fluid	1 (0.3)	0	1 (0.2)	0	2 (0.1)
Total isolates per quartile (n)	333	513	439	252	1,537^*f*^

^*a*^ Percentages of *Achromobacter *isolates by specimen source per quartile are listed in parentheses (%). Sample totals by specimen source are listed in the right column, whereas sample totals by quartile of years are listed in the bottom row.

^*b*^ Includes respiratory samples not obtained through bronchoscopy with bronchoalveolar lavage.

^*c*^ Includes samples obtained from the sinuses.

^*d*^ Includes samples from left ventricular assist devices (LVADs).

^*e*^ Includes abdominal wall skin swabs and tissue samples.

^*f*^ Eight isolates were excluded from this table as they were reported to be from “Other” or unknown sources. Gray cells indicate total numbers of isolates per source or per quartile.

### Antimicrobial susceptibility testing (non-bloodstream isolates)

For antimicrobial susceptibility testing, between 1,000 and 1,250 isolates were tested for most antibiotics (Table 6). Overall, susceptibility was high to piperacillin-tazobactam (92.8%), TMP-SMX (92.1%), meropenem (87.5%), and imipenem (94.6%). Susceptibility was lower to ceftazidime (72.9%) and markedly low to cefepime (11.6%). Resistance to fluoroquinolones and aminoglycosides was significant across all tested isolates (Table 6). With respect to multidrug resistance, defined as resistance to ≥3 classes of antimicrobials, by yearly quartile, 228 of 335 (68.1%) isolates from 2013 to 2015 were multidrug resistant (MDR); 230 of 516 (44.6%) isolates from 2016 to 2018 were MDR; 252 of 441 (57.1%) isolates from 2019 to 2021 were MDR; and 127 of 253 (50.2%) isolates from 2022 to 2023 were MDR.

**TABLE 6:** Susceptibility of all tested non-bloodstream *Achromobacter* isolates per quartile (number of isolates tested [% susceptible isolates])^*a*^

Antibiotic	2013–2015	2016–2018	2019–2021	2022–2023	Total
*Aminoglycosides*					
Amikacin	266 (16.2)	244 (19.3)	328 (10.4)	195 (9.7)	*1,033 (13.8)*
Gentamicin	266 (9.0)	238 (13.0)	327 (8.9)	193 (6.2)	*1,024 (9.4)*
Tobramycin	266 (12.0)	241 (17.0)	325 (9.8)	193 (7.8)	*1,025 (11.7)*
*Fluoroquinolones*					
Ciprofloxacin	267 (6.7)	240 (11.3)	325 (6.8)	193 (5.2)	*1,025 (7.5)*
Levofloxacin	266 (40.6)	285 (44.9)	327 (30.6)	193 (34.7)	1,071 (37.6)
*Tetracyclines*					
Doxycycline	1 (0.0)	2 (50.0)	5 (0.0)	4 (75.0)	12 (33.3)
Minocycline	9 (100.0)	7 (100.0)	16 (62.5)	19 (78.9)	**51 (80.4)**
Tetracycline	2 (0.0)	1 (0.0)	3 (0.0)	1 (0.0)	7 (0.0)
*Cephalosporins*					
Ceftazidime	268 (72.4)	355 (83.1)	326 (66.9)	193 (64.8)	1,142 (72.9)
Cefepime	266 (11.3)	246 (18.7)	326 (9.5)	193 (6.7)	*1,031 (11.6)*
*Carbapenems*					
Meropenem	275 (85.1)	405 (91.4)	358 (84.6)	208 (88.0)	**1,246 (87.5)**
Imipenem	13 (92.3)	24 (100.0)	23 (91.3)	14 (92.9)	**74 (94.6)**
*Other antibiotics*					
Aztreonam	107 (0.9)	244 (1.6)	343 (0.3)	206 (0.5)	*900 (0.8)*
Trimethoprim-sulfamethoxazole	269 (91.8)	381 (92.7)	328 (91.8)	193 (91.7)	**1,171 (92.1)**
Piperacillin-tazobactam	277 (91.7)	406 (96.1)	346 (89.3)	205 (93.7)	**1,234 (92.8)**
Ticarcillin-clavulanic Acid	19 (10.5)	–	–	–	19 (10.5)

^*a*^ Bold face denotes total susceptibility of 80% or greater to the tested antibiotic with at least 30 isolates tested, while Italics text denotes total resistance rate of 80% or greater with at least 30 isolates tested. –, indicates that Data were not available for ticarcillin-clavulanic acid from 2016 to 2023.

A separate analysis was performed for *A. xylosoxidans* pulmonary isolates (Table 7). As compared to bloodstream isolates, pulmonary isolate resistance was significantly higher for ciprofloxacin (84.5% vs 68.8%; p = 0.049) and levofloxacin (49.4% vs 16.0%; p = 0.001), with trends towards higher resistance in TMP-SMX (11.6% vs 3.1%; p = 0.22) and cefepime (82.8% vs 68.8%; p = 0.09) (Table 8).

**TABLE 7:** Susceptibility of *Achromobacter xylosoxidans* pulmonary isolates

Antibiotic	No. of isolates	% susceptible (MIC)	% intermediate (MIC)	% resistant (MIC)
Amikacin^*a*^	243	4.5 (≤16)	6.2 (32)	*89.3 (≥64)*
Aztreonam	214	0 (≤8)	0 (16)	*100 (≥32)*
Cefepime	239	2.1 (≤8)	15.1 (16)	*82.8 (≥32)*
Ceftazidime	241	58.5 (≤8)	13.7 (16)	27.8 (≥32)
Ciprofloxacin	239	2.9 (≤1)	12.6 (2)	*84.5 (≥4)*
Gentamicin	240	2.9 (≤4)	4.2 (8)	*92.9 (≥16)*
Imipenem	27	100 (≤4)	0 (8)	0 (≥16)
Levofloxacin	241	22.0 (≤2)	28.6 (4)	49.4 (≥8)
Meropenem	278	**80.2 (≤4)**	7.2 (8)	12.6 (≥16)
Minocycline	16	68.8 (≤4)	12.3 (8)	18.9 (≥16)
Piperacillin-tazobactam	264	**84.1 (≤16/4)**	6.4 (32/4-64/4)	9.5 (≥128/4)
Tobramycin	239	6.3 (≤4)	5.0 (8)	*88.7 (≥16)*
TMP-SMX	242	**88.4 (≤2/38)**		11.6 (≥4/76)

^*a*^ Bold face denotes susceptibility of 80% or greater to the tested antibiotic with at least 30 isolates tested, while Italics text denotes resistance rates of 80% or greater with at least 30 isolates tested.

**TABLE 8:** Comparison of resistance rates between *Achrombacter xylosoxidans* bloodstream and pulmonary isolates (fraction of tested isolates resistant [% resistant])

Antibiotic	Blood (%)	Pulmonary (%)	*P*-value
Amikacin	30/32 (93.8)	217/243 (89.3)	0.75
Aztreonam	23/23 (100)	214/214 (100.0)	1.0
Cefepime	22/32 (68.8)	198/239 (82.8)	0.09
Ceftazidime	5/27 (18.5)	67/241 (27.8)	0.42
Ciprofloxacin	22/32 (68.8)	202/239 (84.5)	0.049
Gentamicin	31/32 (96.9)	223/240 (92.9)	0.70
Imipenem	1/7 (14.3)	0/27 (0)	0.21
Levofloxacin	4/25 (16.0)	119/241 (49.4)	0.001
Meropenem	1/21 (4.8)	35/278 (12.6)	0.48
Piperacillin-tazobactam	1/32 (3.1)	25/264 (9.5)	0.33
Tobramycin	31/32 (96.9)	212/239 (88.7)	0.22
TMP-SMX	1/32 (3.1)	28/242 (11.6)	0.22

**Fig 1:**
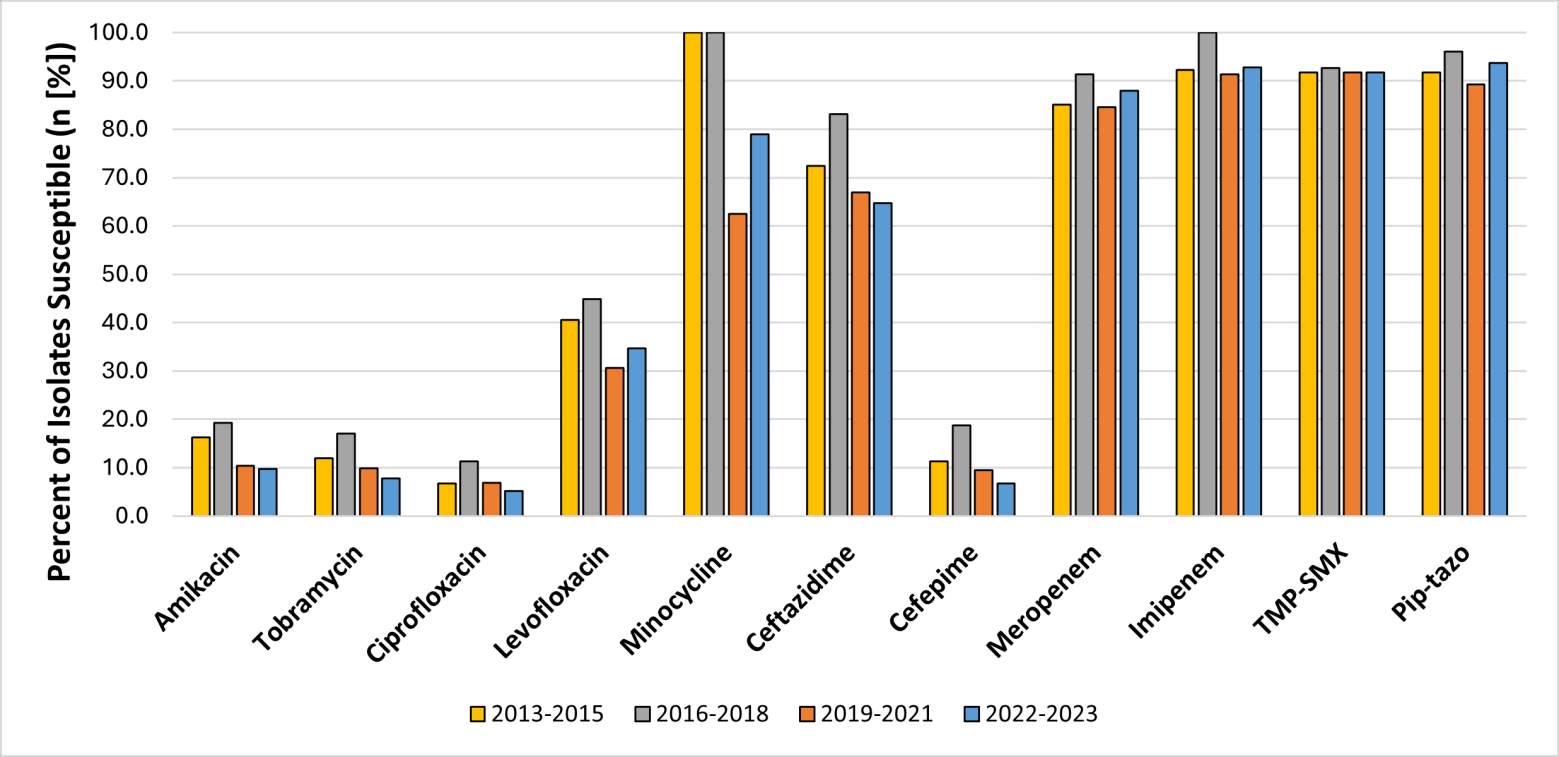
Antibiotic susceptibility of non-bloodstream *Achromobacter* isolates 2013–2023

Over the study period, susceptibility patterns remained stable for piperacillin-tazobactam, TMP-SMX, and carbapenems (Table 6, Fig. 1). Susceptibility decreased for several antibiotics from 2013 to 2015 to 2022–2023, including levofloxacin (40.6% to 34.7%), ceftazidime (72.4% to 64.8%), and minocycline (100% to 78.9%) (Table 6, Fig. 1). For these three antibiotics specifically, the differences were not statistically significant on pairwise comparison (Fisher’s exact *P* > 0.05 for all).

## DISCUSSION

This 10-year, multicenter analysis of *Achromobacter* infections, encompassing 1,545 non-bloodstream and 53 bloodstream isolates, represents the largest study to date that combines both clinical and microbiologic data on this genus. Key findings include high (>85%) susceptibility among both bloodstream and non-bloodstream isolates to meropenem, piperacillin-tazobactam, and TMP-SMX, respiratory isolates of *A. xylosoxidans* exhibiting higher resistance rates to several antibiotics compared to bloodstream isolates, and central venous catheters as the most common potential source of bloodstream infections in our studied cohort.

Antibiotic susceptibility data from this study reinforce prior data suggesting that piperacillin-tazobactam, carbapenems, and TMP-SMX should be considered first-line agents for *Achromobacter* infections (8, 13). However, selection of therapy should account for infection severity, site, and the need for source control, particularly in catheter-associated bloodstream infections. TMP-SMX should be used cautiously in severe infections, given the paucity of data supporting its efficacy in this setting and the risk of toxicity in critically ill patients (8). *Achromobacter* susceptibility rates to ceftazidime fell among non-bloodstream isolates during our study period (64.8% by the end of the study period), and susceptibility to bloodstream isolates (81.8%) was lower than that of piperacillin-tazobactam (92.3%), meropenem (94.7%), imipenem (83.3%), and TMP-SMX (96.2%) (16, 21). Our data thus suggest it may be safest to regard ceftazidime as a less preferred, alternative agent for initial treatment of *Achromobacter* BSI while results of AST are still in process.

When treating respiratory isolates of *A. xylosoxidans* specifically*,* our data suggest that meropenem, piperacillin-tazobactam, and TMP-SMX remain viable initial options, with the caveat that higher rates of resistance were observed for respiratory isolates (with significantly higher resistance to fluoroquinolones), which likely reflects a higher burden of anti-microbial exposure in this cohort. A final point regarding antibiotic selection for *Achromobacter* infections is that patients with CF are likely to require a more nuanced approach to antimicrobial selection given the unique clinical and microbiologic features of CF and likelihood of prior exposure to multiple antibiotics (8).

Within the bloodstream infection cohort, infection was predominantly observed in immunocompromised patients (59.6% of cases). The presence of a central venous catheter in 73.1% of cases underscores its role as a likely source of infection and highlights the importance of catheter management in *Achromobacter* bacteremia. All-cause mortality rates at 30 and 90 days were 10.2% and 18.4%, respectively. These rates are lower than those reported for bacteremia due to *Klebsiella pneumoniae* or other carbapenem-sensitive Gram-negative bacilli (32, 33).

Median duration of antibiotic therapy for BSI was 16 days in this study, and several patients in our cohort received prolonged antibiotic courses (>4 weeks) due to complex infectious pathology and/or limited ability to achieve source control. Patients had a low bloodstream infection relapse rate (1 in 49). There has been a recent shift from 14 days to 7 days (or durations between 7 and 14 days) to complete treatment for Gram-negative bacteremia, with recent studies suggesting shorter antibiotic courses for gram-negative bacteremia are appropriate, even in patients with underlying immunocompromise (34–37). For *Achromobacter* BSI specifically, the optimal treatment duration is unknown and requires further study.

Four patients in our bloodstream infection cohort received combination antibiotic therapy targeted towards *Achromobacter* in their treatment regimen. Although all four of these patients survived the index hospitalization, outcomes data are difficult to compare with the rest of the BSI cohort due to the small number of cases. The role of combination antibiotic therapy in the treatment of *Achromobacter* infections is not well studied, with data limited to case reports/series and *in vitro* analysis (16, 38, 39).

A consistent finding in our study was the high proportion of *Achromobacter* isolates that could not be identified at the species level. This was observed with 53.9% of non-bloodstream isolates and with 34.6% of BSI cases. The percentage of non-bloodstream isolates that were not identifiable at the species level did not fall during the study period and, in fact, was at its highest in the final quartile of the 10-year period. Prior to the advent of MALDI-TOF MS, which has allowed accurate identification of *Achromobacter* on the genus level, *Achromobacter* spp. were often misidentified by traditional laboratory techniques as other nonfermenting Gram-negative bacilli, such as *Pseudomonas aeruginosa* or *Burkholderia cepacia* complex (7, 8). Even with MALDI-TOF MS technology, species-level identification of *Achromobacter* isolates continues to prove challenging, as standard MALDI-TOF MS databases are often incapable of identifying individual *Achromobacter* species and are prone to misidentifying a given isolate as the incorrect *Achromobacter* species (8, 9, 40, 41). In our study, it is likely that improvements in *Achromobacter* taxonomy and the expansion of MALDI-TOF MS databases with time explain much, if not most, of the observed increase in non-speciated *Achromobacter* isolates and the decrease in *Achromobacter xylosoxidans* identification during the study period, rather than there being a true change in species prevalence. Earlier versions of MALDI-TOF MS libraries included only a limited number of *Achromobacter* species, so many non-*xylosoxidans* isolates were misidentified, often by being labeled *A. xylosoxidans* (40). With database improvements over time, the likelihood of mislabeling has decreased, and non-speciated *Achromobacter* isolate reporting has consequently increased.

### Limitations

This study has limitations. Treatment or clinical management data were not collected for non-bloodstream infections. As such, our study was not designed to determine whether non-bloodstream *Achromobacter* isolates represented true infections versus colonization or contamination. Consequently, our findings cannot be used to guide decisions regarding whether to treat non-bloodstream infections. Among bloodstream isolates, only one case was not treated with antibiotics due to being considered a contaminant, suggesting that true infections were well captured in this cohort.

Additionally, this study was conducted in tertiary care centers, introducing the potential for referral bias. Patients at these institutions may have had more complex infections or prior antibiotic exposure, which could influence susceptibility patterns. The generalizability of these findings to community hospitals or populations with different demographics remains uncertain.

Finally, lack of established MIC susceptibility breakpoints for *Achromobacter* spp. and use of inconsistent breakpoints for *Achromobacter* in previous studies can limit comparison of our AST data to that presented by other studies or to future studies. There have been recent efforts to establish MIC breakpoints for *Achromobacter*, and we believe that further research of this topic is much needed and will prove beneficial for future study of *Achromobacter* (29).

### Conclusions

Findings from this 10-year, single-institution analysis support the use of piperacillin-tazobactam, carbapenems, or trimethoprim-sulfamethoxazole as initial therapy for *Achromobacter* infections. However, treatment decisions should be tailored to individual patient factors, particularly in those with cystic fibrosis or other conditions that may require alternative antimicrobial strategies. Further research is needed to clarify the impact of specimen source on antimicrobial susceptibility, monitor evolving resistance patterns, and define optimal treatment approaches for *Achromobacter* infections, including the role of combination antibiotic therapy.

## SUPPLEMENTAL MATERIAL

Table S1 jcm.00724-25-s0001.docx Non-*Achromobacter* pathogens implicated in polymicrobial bloodstream infection (total number of polymicrobial cases = 25).

ASM does not own the copyrights to Supplemental Material that may be linked to, or accessed through, an article. The authors have granted ASM a non-exclusive, world-wide license to publish the Supplemental Material files. Please contact the corresponding author directly for reuse.
