# Supplementary material for: Clinical and microbiologic features of Achromobacter species: a 10-year, multicenter experience
Source: J Clin Microbiol. 2025 Sep 19;63(10):e00724-25. doi: 10.1128/jcm.00724-25 (PMC12505881; doi:10.1128/jcm.00724-25)
Supplement: Table S1 — Non-Achromobacter pathogens implicated in polymicrobial bloodstream infection (total number of polymicrobial cases = 25). [file jcm.00724-25-s0001.docx]

**Supplementary Table 1** Non-*Achromobacter* pathogens implicated in polymicrobial bloodstream infection (total number of polymicrobial cases = 25).

| **Pathogen** | **Number of cases** |
| --- | --- |
| Coagulase-negative staphylococci | 6 |
| *Pseudomonas aeruginosa* | 4 |
| *Stenotrophomonas maltophila* | 4 |
| *Acinetobacter* species | 4 |
| *Enterobacter cloacae/cloacae* complex | 3 |
| *Klebsiella pneumoniae/pneumoniae* complex | 2 |
| *Klebsiella oxytoca* | 2 |
| *Brevibacterium* species | 2 |
| *Enterococcus faecium* | 1 |
| *Staphylococcus aureus* | 1 |
| *Candida glabrata* | 1 |
| *Chryseobacterium hominis* | 1 |
| *Bacillus cereus/thuringiensis* | 1 |
| *Serratia marcescens/ureilytica* | 1 |
| *Elizabethkingia meningoseptica* | 1 |
| *Mycobacterium fortuitum* group | 1 |
| *Micrococcus* species | 1 |
| *Rhizobium* species | 1 |
| *Camelimonas* species | 1 |
| *Microbacterium* species | 1 |
| *Ochrobactrum* species | 1 |
